# The APEX Quantitative Proteomics Tool: Generating protein quantitation estimates from LC-MS/MS proteomics results

**DOI:** 10.1186/1471-2105-9-529

**Published:** 2008-12-09

**Authors:** John C Braisted, Srilatha Kuntumalla, Christine Vogel, Edward M Marcotte, Alan R Rodrigues, Rong Wang, Shih-Ting Huang, Erik S Ferlanti, Alexander I Saeed, Robert D Fleischmann, Scott N Peterson, Rembert Pieper

**Affiliations:** 1 Pathogen Functional Genomics Resource Center, J. Craig Venter Institute, 9704 Medical Center Drive, Rockville, MD 20850, USA; 2 Center for Systems and Synthetic Biology, Department of Chemistry and Biochemistry, Institute for Cellular and Molecular Biology, 2500 Speedway, University of Texas, Austin TX 78712, USA

## Abstract

**Background:**

Mass spectrometry (MS) based label-free protein quantitation has mainly focused on analysis of ion peak heights and peptide spectral counts. Most analyses of tandem mass spectrometry (MS/MS) data begin with an enzymatic digestion of a complex protein mixture to generate smaller peptides that can be separated and identified by an MS/MS instrument. Peptide spectral counting techniques attempt to quantify protein abundance by counting the number of detected tryptic peptides and their corresponding MS spectra. However, spectral counting is confounded by the fact that peptide physicochemical properties severely affect MS detection resulting in each peptide having a different detection probability. Lu *et al*. (2007) described a modified spectral counting technique, Absolute Protein Expression (APEX), which improves on basic spectral counting methods by including a correction factor for each protein (called *O*_*i *_value) that accounts for variable peptide detection by MS techniques. The technique uses machine learning classification to derive peptide detection probabilities that are used to predict the number of tryptic peptides expected to be detected for one molecule of a particular protein (*O*_*i*_). This predicted spectral count is compared to the protein's observed MS total spectral count during APEX computation of protein abundances.

**Results:**

The APEX Quantitative Proteomics Tool, introduced here, is a free open source Java application that supports the APEX protein quantitation technique. The APEX tool uses data from standard tandem mass spectrometry proteomics experiments and provides computational support for APEX protein abundance quantitation through a set of graphical user interfaces that partition thparameter controls for the various processing tasks. The tool also provides a Z-score analysis for identification of significant differential protein expression, a utility to assess APEX classifier performance via cross validation, and a utility to merge multiple APEX results into a standardized format in preparation for further statistical analysis.

**Conclusion:**

The APEX Quantitative Proteomics Tool provides a simple means to quickly derive hundreds to thousands of protein abundance values from standard liquid chromatography-tandem mass spectrometry proteomics datasets. The APEX tool provides a straightforward intuitive interface design overlaying a highly customizable computational workflow to produce protein abundance values from LC-MS/MS datasets.

## Background

The field of proteomics has used mass spectrometry (MS) techniques to provide qualitative results that describe the protein complement of complex protein samples [1]. Researchers also use modifications of these MS technologies for the quantitative analysis of proteins in complex samples [1-3], and often hundreds to thousands of proteins are quantified per experiment. Some quantitative techniques involve peptide isotopic labeling [4-8]. In contrast, label-free techniques have focused on analysis of MS/MS peak heights or observed peptide spectral count information [9-12]. Peptides are produced in an enzymatic digestion of the protein mixture, often using trypsin, which generally cleaves the proteins at the C-terminus of lysine or arginine amino acid residues [13].

Spectral counting techniques typically infer the relative quantity of a protein by counting the number of MS detected tryptic peptides associated with the protein being quantified as a fraction of all observed peptide counts. However, spectral counting can be confounded by the fact that the likelihood of peptide detection by MS techniques can vary greatly from one peptide to another based on the particular physicochemical properties of the peptide sequences. Peptide physicochemical properties can affect final MS detection through several factors such as the ability to recover peptides during the cation exchange and reversed phase LC stages of sample preparation, variation in ionization efficiency of the peptide in the ion source of a particular MS instrument, and can affect mass analysis in MS and MS/MS modes [9,14,15]. Peptide properties such as peptide length, mass, amino acid composition, solubility, net charge, and other properties can impact peptide detection. This variability in peptide detection can lead to errors in assessing the abundance of the parent protein producing the tryptic peptides.

Lu *et al*. [16] have described a novel technique for protein quantitation, Absolute Protein Expression measurements (APEX), where machine learning techniques are used to improve quantitation results over basic spectral counting. In the APEX technique, a supervised classification algorithm is used to predict the probability of peptide detection by MS based on the peptide's physicochemical properties. For each protein in the sample, the expected number of peptide observations (spectral counts) is computed based on predicted MS detectability of the corresponding tryptic peptides. In other words, the computationally predicted (expected) spectral counts are corrected for the variable peptide detection probabilities related to peptide physicochemical properties and the specific MS technology in use.

More formally, the APEX technique, given by equation 1 [16], is a modified spectral counting method in which the total *observed *spectral count for protein *i *(*n*_*i*_) is normalized by a computationally *predicted *or *expected *count (*O*_*i*_) for one molecule of protein *i*. The computed values are weighted based on the protein identification probability (*p*_*i*_). A relative APEX score is obtained by dividing by the sum of the values for all *N *proteins being quantified. The user-supplied normalization factor *C*, typically an estimate of total protein concentration, converts the relative abundance values into absolute terms.

(1) $$APEX_{i}=\frac{p_{i}\left(\frac{n_{i}}{O_{i}}\right)}{\sum_{k=1}^{N}p_{k}\left(\frac{n_{k}}{O_{k}}\right)}\times C$$

APEX abundance estimates are *absolute *in the sense that they are not relative to a second dataset representing a different condition or control, as is done in some relative protein quantitation methods such as SILAC [8]. Also, the abundance estimates within a sample are normalized and can be readily compared to estimates from other samples. While a particular protein's abundance is presented *relative *to all proteins within the sample, multiplication by C puts the abundance values into absolute terms.

This paper describes a new software tool, the APEX Quantitative Proteomics Tool, an implementation of the APEX technique for the quantitation of proteins based on LC- MS/MS proteomics results. The main role of the tool is to compute APEX protein abundance values using equation 1, however the tool also supports preparation of prior information, such as derivation of *O*_*i *_values for proteins under study, as well as post-processing data analysis.

**Figure 1:**
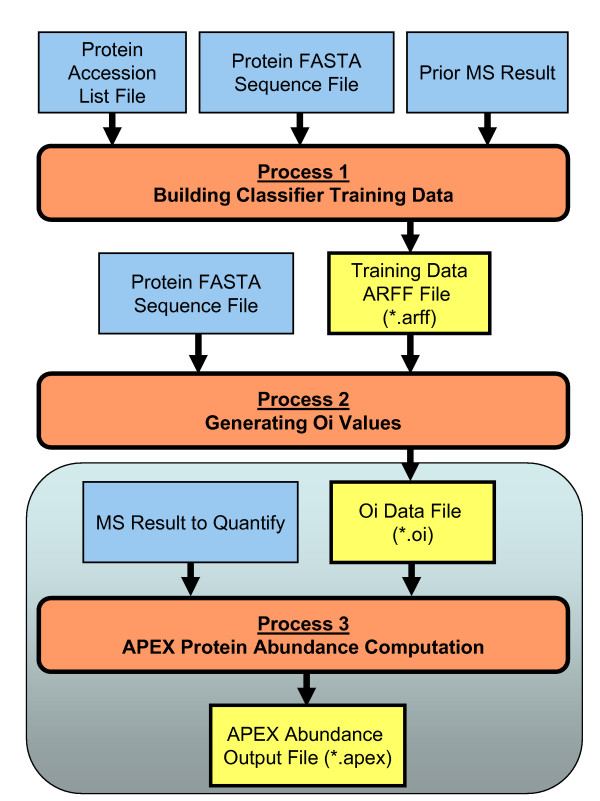
**Primary Processing Tasks within the APEX Quantitative Proteomics Tool**. The flowchart illustrates the three major processing tasks within the APEX tool. Processes are depicted in rounded rectangles while data inputs are shown as rectangles where bold rectangles indicate data that is generated by the APEX tool. The first two processes have the objective to produce *O*_*i *_values while the last process, enclosed in the larger rounded rectangle, uses these *O*_*i *_values to quantify proteins based on the supplied MS results imported in protXML format. Note that *O*_*i *_values only need to be generated once to support quantitation of proteins from the particular proteome under study. Once created, these *O*_*i *_values can be used repeatedly for the same proteome analyzed on the same MS instrument.

The APEX tool supports three primary processing tasks as shown in figure 1. The first task is the construction of a training data set that relates prior peptide MS data to a set of peptide physicochemical properties which is used to predict peptide MS detection probabilities. The peptide MS detection probabilities are needed to estimate expected spectral counts for each protein (*O*_*i *_values). The use of prior (user-defined) MS data insures that the later calculations reflect the specific laboratory protocols, MS instrumentation, instrument settings, and the particular proteins under study and other factors that could influence peptide detection. The training data can be an independent high-quality MS dataset or even an experimental dataset.

The second processing task is the generation of an *O*_*i *_value for each protein under study. This step uses the generated training data, peptide physicochemical properties and peptide MS detection calls, to build a classifier to predict peptide detection probabilities. Each protein sequence from a supplied FASTA sequence file undergoes an *in silico *trypsin digestion and each peptide is assigned an MS detection probability. The probabilities for each peptide derived from protein *i *are summed to produce the protein's *O*_*i *_value. This *O*_*i *_value is the predicted peptide detection (spectral) count for one molecule of protein *i*.

The third processing task uses the previously generated *O*_*i *_values and LC-MS/MS experimental results, which provide *n*_*i *_and *p*_*i*_, to produce protein abundance values according to equation 1. These quantitation results can be piped into several post-processing tools.

## Implementation

### Building Training Data

**Figure 2:**
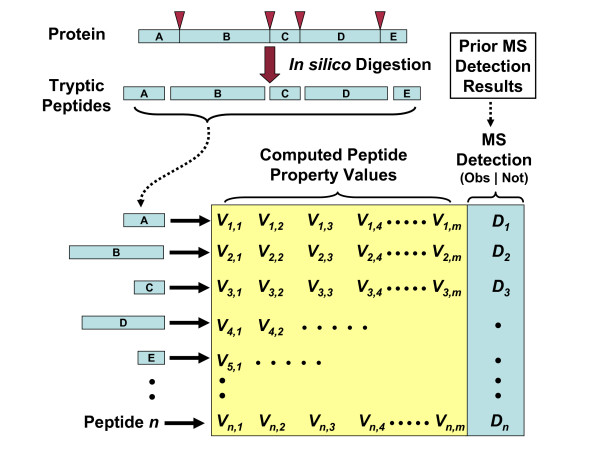
**Building Classifier Training Data**. This diagram describes the construction of training data. A set of protein sequences undergo a trypsin *in silico *digestion to form a collection of tryptic peptides. A number (*m*) of peptide physicochemical properties are computed for each peptide (for peptide *i*, properties 1-m are denoted in the figure as: V_i,1_, V_i,2_, V_i,3_,... V_i,m_,) and prior MS results are searched to determine if the peptide has been observed or not (for peptide *i*, the detection call is denoted in the figure as D_*i*_). The resulting training data forms a matrix of values where each row represents the values related to a particular peptide. This output training data associates peptide properties with the MS detection call and will later train a classifier to produce peptide detection probabilities based on peptide physicochemical properties.

The construction of training data, diagramed in figure 2, involves an initial *in silico *trypsin digestion of a selected set of protein sequences, enabling the generation of a species- or condition-specific training dataset. Prior MS results are used to provide information on whether the tryptic peptides are observed or not. The input protein set represents a collection of proteins that are most likely to be correctly identified by MS. The particular proteins selected for input are not critical; however, they should be proteins that are present in the input MS result and give rise to tryptic peptides that have physicochemical properties that vary and result in a mix of observed and non-observed peptides. These input proteins are specified by a protein accession list and the corresponding protein sequences are supplied in a standard FASTA format file. The selection of a training dataset relies on the fact that even for high abundance proteins with high confidence identifications (as often specified in the input protein accession list) some peptides are observed in the MS/MS data, whereas others are not. The observation and non-observation of these peptides is used for training. The types and identities of the training proteins (peptides) are independent of the experimental data that is to be analyzed later; however, they should be selected to represent an unbiased representation of peptide detection given the specific MS conditions. Some of the peptide sequences derived from *in silico *digestion of the input proteins may be generated from more than one parent protein. These peptides are termed degenerate peptides and the current implementation insures that peptides within the training set are unique to deal with the possibility that more than one protein may give rise to them during digestion. The observation or lack of observation of these degenerate peptides in prior MS results is dependent on the nature of the peptide. The particular parent protein or proteins giving rise to the peptide is not of consequence to peptide detection by MS.

Several peptide physicochemical properties are computed for each of the corresponding tryptic peptides; the APEX tool supports the computation of as many as 35 different properties. Among these properties are peptide mass, length, amino acid composition, and properties related to charge, hydrophobicity measures, and amino acid frequencies within secondary peptide structures. The value of each peptide property, in terms of predicting whether a peptide will be observed by MS, varies based on the MS technology in use [14]. The 35 peptide properties available in the APEX tool are a combination of properties identified in the APEX technique paper by Lu *et al*. [16] and of those described in the paper by Mallick *et al*. [14]. The list of peptide properties can be found in the APEX manual's Appendix.

Next, prior MS result files in standard protXML format are searched for each tryptic peptide sequence and each tryptic peptide is given a *peptide MS detection call *which categorizes it as being either *observed *or *not observed within the MS result*. The input protXML MS result files are generated by preprocessing standard SEQUEST or Mascot files using PeptideProphet™ and ProteinProphet™ which are part of the Trans-Proteomic Pipeline (TPP) [17,18]. Once the peptide MS detection calls have been determined, the data is output in a matrix format as depicted in figure 2. Each row in the matrix captures data related to a single peptide and includes a set of computed peptide physicochemical properties and the peptide MS detection call. The training data is output to a file in the Attribute-Relation File Format (ARFF). The ARFF format represents the matrix of training values in a comma delimited format and has a section that identifies the attributes or columns in the data matrix. The ARFF format is used as input by the Weka collection of machine learning data mining tools [19,20]. The generated training data file merges peptide properties and peptide MS detection calls, and will be used to train a classifier to compute peptide detection probabilities based on peptide physicochemical properties.

### O_i _Value Generation

**Figure 3:**
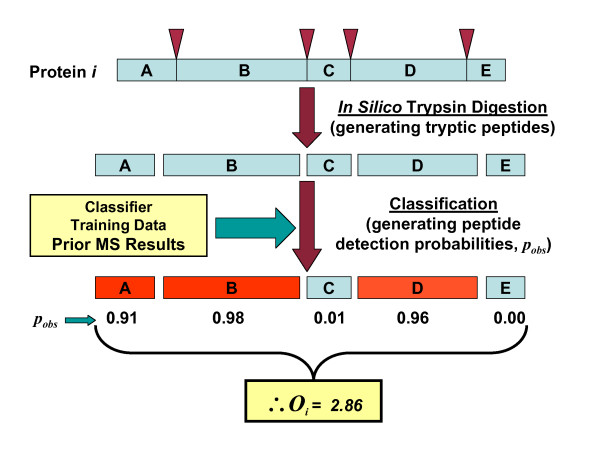
**Computing Oi Values**. This figure provides an overview of Oi generation. Each protein undergoes an *in silico *trypsin digestion to form peptide sequences that are fed into a classifier trained on previously constructed training data. The classifier generates peptide detection probabilities. Peptide probabilities related to a particular protein *i *are summed to arrive at the protein's *Oi *value, or predicted detected spectral count for one instance of the protein.

In the second processing step, the APEX tool calculates *O*_*i *_values for all proteins under study using the training ARFF file created in process 1 and sequences of the proteins of interest. The protein sequences are input in a FASTA format file. They undergo an *in silico *trypsin digestion and peptide properties are computed for each peptide. The training data is used to train a classifier which generates individual peptide detection probabilities based on peptide properties. For each particular protein *i*, the *O*_*i *_value is the summation of all peptide detection probabilities for the peptides derived from protein *i*. This value estimates the number of peptides derived from one molecule of protein *i *that will be observed by MS/MS analysis. Figure 3 illustrates the *O*_*i *_generation process.

The classification algorithms in the APEX tool are implementations from the Weka data mining software package. Weka is an extensive collection of open source machine learning algorithms implemented in Java [19,20]. The APEX tool allows the user to select from three different Weka classifier algorithms: Random Forest, RIDOR (Ripple Down Rule Learner), and J4.8 Decision Trees. The original work on the APEX technique [16] showed that averaging classifier models through bootstrap aggregating (Bagging) improved classifier performance [16]. This work also found performance improvements when building the classifier as a cost sensitive classifier to account for the bias in the training data toward non-observed peptides; training data peptides were not evenly split between observed and non-observed such that non-observed peptides are more prevalent. The APEX tool provides both the option to perform classifier algorithms using bagging and cost sensitive evaluation. Although the APEX tool provides three classifier options by default, the tool also includes a classifier configuration file that can be edited to allow one to configure the APEX tool to make use of any classifier algorithm implemented in the Weka tool set. The classifier configuration file lists the available classifiers and defines the unique parameter attributes for each classifier. Random Forest is the default classification algorithm within the APEX tool since this algorithm had been found to perform best [16]. The Random Forest classifier has worked well in our evaluation. The tool has a utility to permit users to evaluate classifier performance in the context of their own data.

### Computing APEX Quantitation Values

The third and primary processing task of the APEX tool uses the *O*_*i *_values and an MS data file in protXML format as input to generate abundance values for each protein according to equation 1. The MS protXML file supplies protein identification probabilities (*p*_*i*_) and spectral counts (*n*_*i*_). During execution, the input protein set from the protXML file is presented in a list in decreasing *p*_*i *_rank so that protein identifications of highest confidence are displayed first. The *p*_*i *_values are used to compute a false positive rate (FPR) for any selected subset of the protein list according to equation 2 [21] where *k *represents the number of selected proteins from the full list of input proteins in the input file sorted by *p*_*i*_.

(2) $$FPR=\frac{1}{k}\sum_{i=1}^{k}\left(1-p_{i}\right)$$

The FPR can be used to select a subset of high confidence proteins on which to perform APEX quantitation. The APEX tool thereby provides the user with a choice to determine the cutoff FPR for APEX quantitation, typical cutoffs are 1 or 5%. Following the selection of the protein list, an output file with the APEX quantitation results is generated. The output file captures protein identifier or accession, protein descriptive annotation available in the protXML input file, input parameters, input file paths, input MS values (*n*_*i *_and *p*_*i*_), *O*_*i *_values, and the APEX abundance values.

### APEX Tool Implementation Details and Architecture Overview

**Figure 4:**
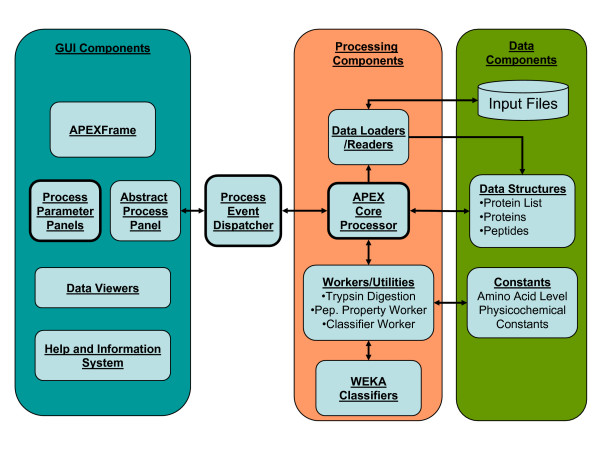
**Schematic Representation of the APEX Tool Software Architecture**. This figure shows the organization of the APEX java package organization. Java classes and resources are divided into those that support the graphical user interface (GUI), those that are responsible for data processing, and those resources responsible for data management such as the primary data structures. Additional processing tasks can be built by adding a new processing panel to the interface, making adjustments to the processing event dispatcher to handle the task, and adding methods to the main processor class to perform the task. Formal UML Class diagrams are available with the APEX source code download.

The APEX tool was coded using Java and therefore is operating system independent. The APEX tool is compatible with computers running Microsoft™ Windows^®^, Linux^®^, and Mac^® ^OSX. The APEX tool software design provides a flexible framework to support future modifications. Figure 4 provides a schematic overview of the APEX tool's architecture and package structure. The primary data structure consists of a three tier system in which a protein list object, APEXProteinList, contains a collection of APEXProtein objects, that in turn can contain a collection of APEXPeptide objects. This simple structure maintains a connection between peptide objects and their parent proteins. Protein and peptide objects serve as container objects for sequence, annotation, and numerical data fields required for APEX computation. Data loaders populate these structures from FASTA or protXML files. Worker or utility classes work on APEXProtein objects to perform tasks such as *in silico *digestion to produce child APEXPeptide objects and they work on APEXPeptides to compute peptide physicochemical properties.

User interface classes are separated from processing classes by the use of a processing event dispatcher that spawns processing threads as needed. Developers can easily add new processing tasks by extending an abstract process panel class that presents parameter controls and by adding a new processing class or adding methods to the core processing class. Constants such as amino acid level physicochemical properties are contained in a single class that contains numerical constants. Unified Modeling Language (UML) class diagrams that cover several of the key Java classes within version 1.0 of the APEX tool are available within the APEX tool source code download.

## Results and discussion

**Figure 5:**
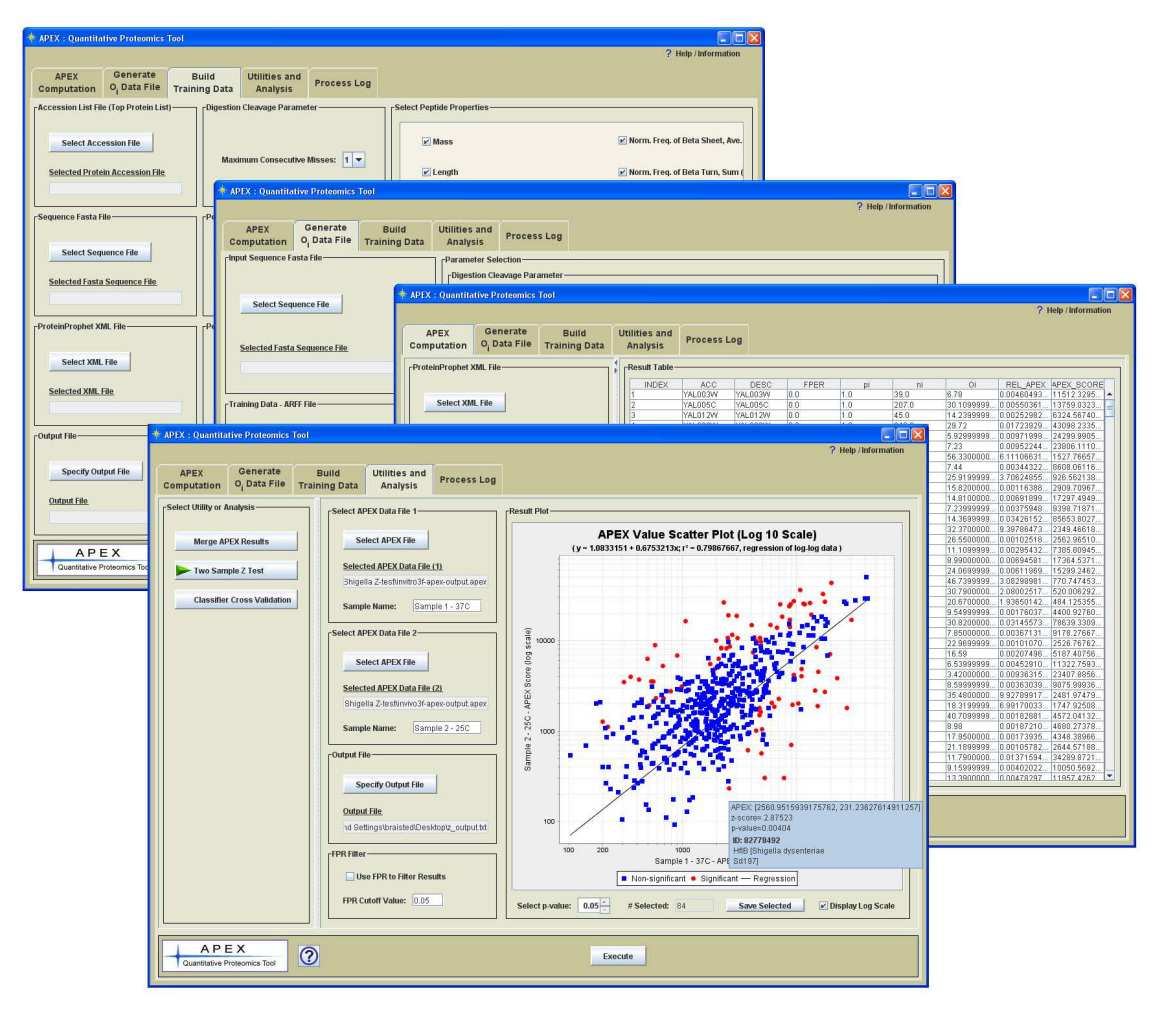
**APEX Tool Interface: Main interface, primary parameter panels and utilities/analysis panel in foreground**. The main interface is composted of a series of tabbed panels. Each panel contains controls for a specific processing task or utility. The user help and information system and tutorial are integrated features that are launched from within the interface via the menu or information buttons.

The APEX Quantitative Proteomics Tool is a free open source Java application for the quantitation of proteins from LC-MS/MS data sets. The APEX tool has a graphical user interface (Figure 5) where the parameter controls for each processing task are encapsulated in a different tabbed panel in the interface. The three major processing tasks, training data construction, generation of *O*_*i *_values, and APEX abundance computation are each handled on separate panels in the interface (Figure 5). Each of these primary processing tasks accepts file and parameter inputs by setting controls on their dedicated parameter panels. The tool has an integrated help system that can be accessed via information buttons on the parameter panels or via the help and information menu. The APEX tool includes a tutorial and sample data to help the user become familiar with the basic use of the tool.

In addition to protein quantitation, the APEX tool also offers basic utilities for post processing of quantitation results (Figure 5, Utilities and Analysis interface panel). One utility merges multiple APEX result files into a tab delimited matrix that contains protein quantitation results. Each data row contains protein annotations and a set of abundance values that represent protein expression over the various conditions under study. Results are aligned so that each row represents a vector of abundance values for a particular protein. The tab delimited data matrix can be loaded directly into the MultiExperiment Viewer (MeV) [22]. MeV contains many methods to cluster proteins based on expression profile and can perform statistical analyses to find proteins showing differential expression in accordance with the experimental conditions.

The APEX tool also provides a two sample Z-score test for differential expression as described in Lu *et al*. [16]. This test handles experimental designs with two samples, each representing a condition or state under study. During the test each protein has a Z-score computed that reflects differential expression by considering the proportion of spectra in the two samples attributable to the protein being scored. Proteins with very different total spectral counts (*n*_*i*_) between the two samples and whose spectral counts are sufficiently large *tend *to have high Z-scores. The formula and underlying assumptions behind the test are given in Lu *et al*. [16]. The Z-score has an associated a p-value for each protein which reflects the significance of the observed expression change by reporting the probability of having an absolute Z-score of the observed magnitude or greater. The APEX interface provides a graphical representation of the Z-score results from the two files to allow the selection of a significant protein lists based on the user defined p-value cutoff (Figure 5, foreground panel). The test outputs a summary result file that contains a row for each protein listing the protein annotations, APEX values for the two conditions under study, APEX abundance fold change, *n*_*i *_values, computed Z-score and p-value.

The third utility provides classifier cross-validation which reports on the performance of the selected classifier and particular parameter selections. This process requires an input training data file and iteratively uses a randomly selected subset of the data to train the classifier and tests the classifier's ability to predict peptide detection calls on the rest of the data. A number of performance statistics are reported, for example true positive rate, false positive rate, prediction accuracy, and recall, that can be saved to a text file. This feature allows to determine which classifiers, classifier parameters, and peptide properties perform best considering the nature of the data and the MS technology in use.

Several potential features are targeted as future enhancements of the APEX tool. Future versions of the tool will include improved support for selecting proteins for the generation of peptide sequences for classifier training data. The training data should include a set of peptides with sufficient representation of observed and non-observed peptides based on prior MS results. The future APEX tool will enable the user to set protein selection criteria such as number of proteins or peptides to include and a minimum p_i _value. The training data selection enhancement will also include randomized selection of training proteins from a larger pool of proteins that pass the imposed criteria. In addition, we will allow users to exclude degenerate peptides that map to more than one parent protein.

Data preprocessing options are another area of future development in the APEX tool. APEX computation requires a protein identification probability (*p*_*i*_). The original APEX methodology paper [16] and this implementation both depend on the Trans-Proteomic Pipeline (TPP) to preprocess MS data to compute the required *p*_*i *_values. Support for TPP derived input will continue but we will expand input options for users not using the TPP for upstream data processing.

Thus far, our data are based on peptide detection and fragmentation in 3D and linear ion trap mass spectrometers (LCQ and LTQ, Thermo Fisher Scientific Inc.). However, the APEX tool is not limited to data processing from these mass analyzers. The training data generation uses prior MS results to insure that the training data reflects the peptide detection capabilities of the instrumentation in use. In turn, *O*_*i *_values generated from the training data will adjust based on peptide detection sensitivity tendencies of the instrument in use. The APEX protocol site [23,24] has posted files containing *Oi *values generated from both LCQ and LTQ-Orbitrap™ MS data for three different organisms, *E. coli*, yeast, and human. The APEX tool can be used to construct training data and generate *O*_*i *_values for data derived from any MS instruments, reflecting characteristics of the individual instruments. Additional peptide properties can be incorporated as they are identified by users as valuable toward improving peptide detection predictions. Future versions of the APEX tool will include a new utility to assess the predictive value of each peptide property given a particular training data set, classifier algorithm and associated parameters. The accuracy of estimated protein abundances depends on the quality of peptide detection probabilities. Further work in this area will refine understanding of peptide properties that are good predictors of peptide detection by particular MS techniques, as an extension of published work [14,15].

## Conclusion

The APEX Quantitative Proteomics Tool provides researchers with the ability to quantify proteins observed in LC-MS/MS proteomics data. This process requires generation of classifier training data and the computation of *O*_*i *_values, *i.e*., the expected spectral counts for each protein. Both the training data and *O*_*i *_values are based on prior MS results that in turn relate to the specific conditions within the user's protocol, including sample preparation procedures, MS technology, and instrumentation settings. Customized *O*_*i *_value generation, facilitated by the APEX Tool, means that the final quantitation values take into account the user's settings and are thus more accurate.

The APEX Tool is a free open source tool and has an intuitive user interface that logically subdivides the controls for the various processing tasks and utilities onto separate tabbed panels. The integrated help and information system and the manual describe both the mechanics of processing data as well as the precise details of how the data is handled at each step of the process. The APEX tutorial provides a step-by-step introduction for the first time user. Source code allows those interested in the computational details to fully explore the inner workings of the tool while the simple software architecture will allow developers to modify or expand on existing utilities.

## Availability and requirements

• Project name: APEX Quantitative Proteomics Tool

• Project Home Page: 

• Operating Systems: Platform Independent

• Programming Language: Java

• Other Requirements: Java 1.5 or higher, Trans-Proteomic Pipeline (TPP) tools to process MASCOT dat files or SEQUEST HTML summary files to produce protXML input files. TPP tools: 

• License: GNU GPL v3.0

• Any restrictions to use by non-academics: None.

## Abbreviations

MS: Mass Spectrometry; LC-MS/MS: Liquid Chromatography with Tandem Mass Spectrometry; APEX: Absolute Protein Expression; ARFF: Attribute Relation File Format; FPR: False Positive Rate.

## Authors' contributions

JCB – software engineering, tool design and development, and primary manuscript author, SK – project conception, provided proteomics domain expertise, researched APEX technique, provided training and test data sets, tested software prototypes, software design, scientific consultation, and manuscript review, CV – an original author of the APEX technique and protocol papers, provided test data sets, support for computational implementation, prototype testing, manuscript review, EMM – an original author of the APEX technique and protocol papers, provided test data sets, support for computational implementation, prototype testing, manuscript review, ARR – researched technique, drafted original Perl implementation, technique evaluation, RW – software design, scientific (proteomics/computational) consultation, SH – input on software design, proteomics domain consultation, manuscript, ESF – support for network upgrades for data preprocessing, software design, AIS – software design, manuscript contributions, RDF – manuscript contributions, project oversight, SNP – manuscript contributions, project oversight, RP – project conception, provided proteomics domain expertise

## References

[B1] Aebersold R, Mann M (2003). Mass spectrometry-based proteomics. Nature.

[B2] Mueller LN, Brusniak M, Mani DR, Aebersold R (2008). An Assessment of Software Solutions for the Analysis of Mass Spectrometry Based Quantitative Proteomics Data. J Prot Res.

[B3] Steen H, Pandey A (2002). Proteomics goes quantitative: measuring protein abundance. Trends Biotech.

[B4] Conrads TP, Issaq HJ, Veenstra TD (2002). New tools for quantitative phosphoproteome analysis. Biochem Biophys Res Commun.

[B5] Mirgorodskaya OA, Kozmin YP, Titov MI, Körner R, Sönksen CP, Roepstorff P (2000). Quantitation of peptides and proteins by matrix-assisted laser desorption/ionization mass spectrometry using ^18^O-labeled internal standards. Rapid Commun Mass.

[B6] Gygi SP, Rist B, Gerber SA, Turecek F, Gelb MH, Aebersold R (1999). Quantitative analysis of complex protein mixtures using isotope-coded affinity tags. Nature Biotech.

[B7] Zhou H, Ranish JA, Watts JD, Aebersold R (2002). Quantitative proteome analysis by solid-phase isotope tagging and mass spectrometry. Nature Biotech.

[B8] Ong SE, Blagoev B, Kratchmarova I, Kristensen DB, Steen H, Pandey A, Mann M (2002). Stable isotope labeling by amino acids in cell culture, SILAC, as a simple and accurate approach to expression proteomics. Mol Cell Proteomics.

[B9] Rappsilber J, Ryder U, Lamond AI, Mann M (2002). Large-Scale Proteomic Analysis of the Human Spliceosome. Genome Res.

[B10] Liu H, Sadygov RG, Yates JR (2004). A model for random sampling and estimation of relative protein abundance in shotgun proteomics. Anal Chem.

[B11] Gao J, Opiteck GJ, Friedrichs MS, Dongre AR, Hefta SA (2003). Changes in the protein expression of yeast as a function of carbon source. J Proteome Res.

[B12] Ishihama Y, Oda Y, Tabata T, Sato T, Nagasu T, Rappsilber J, Mann M (2005). Exponentially modified protein abundance index (emPAI) for estimation of absolute protein amount in proteomics by the number of sequenced peptides per protein. Mol Cell Proteomics.

[B13] Washburn MP, Wolters D, Yates JR (2001). Large-scale analysis of the yeast proteome by multidimensional protein identification technology. Nat Biotech.

[B14] Mallick P, Schirle M, Chen SS, Flory MR, Lee H, Martin D, Ranish J, Raught B, Schmitt R, Werner T, Kuster B, Aebersold R (2007). Computational Prediction of Proteotypic Peptides for Quantitative Proteomics. Nat Biotech.

[B15] Tang H, Arnold RJ, Alves P, Xun Z, Clemmer DE, Novotny MV, Reilly JP, Radivojac P (2006). A computational approach toward label-free protein quantification using predicted peptide detectability. Bioinformatics.

[B16] Lu P, Vogel C, Wang R, Yao X, Marcotte EM (2007). Absolute Protein Expression Profiling Estimates the Relative Contributions of Transcriptional and Translational Regulation. Nat Biotech.

[B17] Keller A, Eng J, Zhang N, Li X, Aebersold R (2005). A uniform proteomics MS/MS analysis platform utilizing open XML file formats. Mol Syst Biol.

[B18] Trans-Proteomics Pipeline. http://tools.proteomecenter.org/TPP.php.

[B19] Witten IH, Frank E (2005). Data Mining: Practical Machine Learning Tools and Techniques with Java Implementations.

[B20] Weka Machine Learning Data Mining Tools. http://www.cs.waikato.ac.nz/ml/weka/.

[B21] Keller A, Nesvizhskii A, Kolker E, Aebersold R (2002). Empirical Statistical Model To Estimate the Accuracy of Peptide Identifications Made by MS/MS and Database Search. Anal Chem.

[B22] TM4 Software Suite's MultiExperiment Viewer. http://www.tm4.org/mev.html.

[B23] APEX Protocol Website, Marcotte Lab. http://www.marcottelab.org/APEX_Protocol.

[B24] Vogel C, Marcotte EM (2008). Calculating Absolute and Relative Protein Abundance from Mass Spectrometry-based Protein Expression Data. Nat Protoc.

